# Non-Contraceptive Benefits of Oral Hormonal Contraceptives

**DOI:** 10.5812/ijem.4158

**Published:** 2012-12-21

**Authors:** Adolf E Schindler

**Affiliations:** 1 Institute for Medical Research and Education, Essen, Germany

**Keywords:** Contraceptives, Oral, Hormonal, Therapeutics, Prevention and Control

## Abstract

**Abstract:**

It is becoming evident that oral hormonal contraceptives-besides being well established contraceptives-seem to become important medications for many functional or organic disturbances. So far, clinical effectiveness has been shown for treatment as well as prevention of menstrual bleeding disorders and menstrual-related pain symptoms. Also this is true for premenstrual syndrome (PMS) and premenstrual disphoric disorder (PMDD).

Particular oral contraceptives (OCs) containing anti-androgenic progestogens were shown to be effective medications for treatment of androgenisation symptoms (seborrhea, acne, hirsutism, alopecia).

Through perfect suppression of the hypothalamic-pituitary-ovarian axis OCs have proven to be effective in elimination of persistent follicular cysts. Endometriosis/adenomyosis related pain symptoms are well handled similar to other drugs like Gonadotropine Releasing Hormone agonists but are less expensive, with less side effects, and possibility to be used for longer periods of time. This is also true for myoma. Pelvic inflammatory disease, rheumatoid arthritis, menstrual migraine, and onset of multiple sclerosis are prevented or delayed. Bone density is preserved and asthma symptoms improved. Endometrial hyperplasia and benign breast disease can be controlled. There is definitely a significant impact on risk reduction regarding endometrial, ovarian, and colon cancers.

In conclusion, it needs to be recognized that oral combined hormonal contraceptives (estrogen/ progestogen combination) are - besides being reliable forms of contraception - are cost-effective medications for many medical disorders in women. Therefore, these contraceptives drugs are important for female and global health and should be used in clinical practice.

## 1. Introduction

Since starting intake of oral hormonal contraceptives as combined estrogen/ progestogen formulation in 1960, these drugs have experienced a steady change in utilized progestogens having different patterns of partial effects in addition to its basic effects. These aspects of progestogen actions have proven to be useful in incorporating by and by non-contraceptive use of these hormonal contraceptives into the list of indications creating a wide range of beneficial effects besides the primary aims. Also, some changes have been implemented in estrogen component. Although OCs started with mestranol and ethinylestradiol, intake of mestranol was abandoned because of its conversion to ethinylestradiol in the body. After about 50 years, recently hormonal contraceptives are further developed by either estradiol valerate or micronized estradiol as estrogen component associated with lowering effect on thromboembolic risk.

Awareness to non-contraceptive benefits of hormonal contraceptives has to be enforced because these hormonal contraceptives besides their undoubtedly high contraceptive efficacy have been shown to be also highly effective in their non-contraceptive benefits. In addition to high therapeutic value in different medical indications, there are very favorable cost/benefit ratio and high level of toleration for these drugs compared to other indicated medications.

Therefore, this paper was aimed to gather and classify different clinical entities associated with cyclic use of oral hormonal contraceptives for effective treatment of various clinical conditions (Table 1). Recently, it was stated that non-contraceptive health benefits were recognized as an important aspect of overall impacts of oral hormonal contraceptives (1).

**Table 1:** Clinical Entities in Which Treatment, Prevention, and Risk Reduction by Hormonal Contraceptives Have Been Shown.

Disorders
**1- Menstrual bleeding disorders**
**Bleeding interval and regularity (poly- and oligomenorrhea)**
**Amount of bleeding (hypermenorrhea/menorrhagia)**
**2-Dysmenorrhea**
**3-Signs of androgenisation (seborrhea, acne, hirsutism, alopecia)**
**4-Premenstrual syndrome (PMS) / Premenstrual dysphoric disorder (PMDD)**
**5-Ovarian cysts**
**6-Endometriosis/adenomyosis**
**7-Myoma**
**8-Pelvic inflammatory disease (PID)**
**9-Rheumatoid arthritis**
**10-Multiple sclerosis**
**11-Menstrual migraine**
**12-Bone structure**
**13-Voice**
**14-Asthma**
**15-Benign breast disease**
**16-Ovarian cancer**
**17-Endometrial hyperplasia /Endometrial cancer**
**18-Colon cancer**

Hypertension defined as current use of antihypertensive drugs or BP more than 140/90 mmHg on two separate occasions Hyperlipidemia: Fasting TG>150 mg per deciliter TC> 240 and or LDL >160mg per deciliter

## 2. Menstrual Bleeding Disorders

Excluding malignancies, menstrual bleeding disorders are common- diseases that can lead to pain, anemia, and loss of quality of life. Indeed, primary oral hormonal contraceptives were approved by the Food and Drug Administration (FDA) for treatment of menstrual disorders (2). None of these therapeutic uses appear in the FDA-approved labeling for currently marketed OCs (3).

Combined estrogen/progestogen preparations are preferentially monophasic drugs of choice for cyclic intakes. Also, extended regimens or constant management are progressively apply applied (4). This improves, on the one hand, the effectiveness and on the other hand, the attractive convenience of such regimens (5, 6).

## 3. Dysmenorrhea

Menstruation associated dysmenorrhea is a relatively frequent event, which can force women to even withdraw from school or work and cause considerable impacts on quality of life. Dysmenorrhea is considered to be the main symptom in women with, but not limited to, endometriosis. Today, dysmenorrheal hormonal contraceptive pills as monophasic estrogen/progestogen combinations are considered as the first choice to subside pain symptoms. In case of insufficient effect, “long-cycle regimen” or constant usage is recommended. The optimal individual effect can be achieved when amenorrhea occurs during which quality of life improves to its best (7).

## 4. Symptoms of Androgenisation (Seborrhea, Acne, Hirsutism, Alopecia)

In general, combined estrogen/progestogen preparations have been useful to treat symptoms of androgenisation because androgens secreted from the ovary will be suppressed and sex hormone binding globulin elevated that in turn will decrease the amount of free, biologically active androgens which reduce the negative action of androgens on sebaceous glands and hair follicles (7, 8). In addition, there are four progestogens, i.e. cyproterone acetate, dienogest, drospirenone, and chlormadinone acetate, with distinct anti-androgenic action by blocking androgen receptors and inhibiting action of circulating androgens. The progestogen with the highest anti-androgenic activity is cyproterone acetate (8). Besides treating effectively the symptoms of androgenisation, there was normalization of the ovaries in structure and size in women with polycystic ovary syndrome (PCOS). The volume of the ovaries as well as the number of follicles decreased. All these effects were highly significant ( 9, 10). Also, these favorable structural changes continued to be present to some extent after the pill cessation. In addition, it was demonstrated that in some of those women, pattern and function of menstrual cycle improved after the treatment (Table 2 and Table 3) (10, 11).

**Table 2:** Changes of Ovarian Volume and Number of Ovarian Follicles Before and After Treatment With 0,35 mg Ethinylestradiol And 2 Mg Cyproterone Acetate According to 9.

Time	Right Ovary, cm^3^	Number of Cystic Follicles	Left Ovary, cm^3^	Number of Cystic Follicles
**Before therapy **	17.3	6.8	18.5	6.3
**During therapy**	8.3	1.2	7.6	1.1
**After therapy**	13.2	5.1	10.7	4.1

**Table 3:** Changes of Ovarian Volume, Number of Cystic Follicles, and Percentage of Women with PCOS Treated for 6 Cycles With 0,035 mg Ethinylestradiol and 2 mg Cyproterone Acetate with Post Therapy Follow-Up of 6 Month According to 10.

Parameter	Baseline	60 Treatment Cycles	6 Month After
**Ovarian volume, cm^3^**	15.1 ± 2.9	6.3 ± 1.1 a	9.5 ± 2.1 b,c
**Number of cystic follicles**	12.1 ± 1.9	1.3 ± 0.7 a	8.2 ± 1.8 b,c
**Stroma, %**	20	50	35

^a^ P ˂ 0,01 versus baseline

^b^ P ˂ 0,05 versus baseline

^c^ P ˂ 0,05 versus 60 treatment cycles

## 5. Premenstrual Syndrome (PMS) and Premenstrual Disphoric Disorder (PMDD)

Both PMS and PMDD disappear during pregnancy and lactation, or after the failure of hypothalamic-pituitary-ovarian axis, or after natural or surgical menopause (12). Some effects were observed primarily by hormonal contraceptives. The introduction of hormonal contraceptives containing drospirenone with a strong anti-mineralocorticoid effect resulted in an effective clinical treatment regimen; the efficiency was increased by an extended regimen of switching from Yasmin® (21/7) to Yaz® (24/4) (7, 12, 13). The latter preparation was recognized by FDA as a treatment modality for PMDD.

## 6. Ovarian Cysts

There is a protection for both follicular and corpus luteum cysts if hormonal contraceptives suppress hypothalamic-pituitary-ovarian axis, properly that depends, for instance, on the type of hormonal contraceptives used as shown in Table 4. This can contribute to avoid the operation (14).

**Table 4:** Changes in Menstrual Cycle Function After 2 Years of Treatment with 0,035 mg Ethinylestradiol and 2 mg Cyproterone Acetate according to 9.

Menstrual Cycle Function	Patients No. (%)
** Prior to therapy **	
Anovulation	28 (84.9)
Oligoovulation	5 (15.1)
** After therapy **	
Ovulation	15 (45.4)
** Menstrual Cycle Length **	** Patients No. (%) **
** Prior to therapy **	
Amenorrhea	4 (12.1)
Oligomenorrhea	22 (66.6)
Eumenorrhea	7 (21.2)
** After therapy **	
Eumenorrhea	14 (42.4)

## 7. Endometriosis/Adenomyosis

Combined estrogen/progestogen pills with a dominant action of progestogen effectively influence on endometriosis-related pain symptoms (15). This treatment can fail for which a so-called long-cycle regimen was introduced (16). However, today, one may start a constant, progestogen-dominant, monophasic combined estrogen/progestogen preparations, instantly. Indeed, the Royal College of Obstetrician Gynecologists recommends combined estrogen/progestogen pill as drug of choice for treating symptoms of endometriosis. This is considered as a safe and economical treatment and as an alternative to surgery (17). This type of therapy not only alleviates pain symptoms but also reduces the size of endometriosis lesions. This effect can be best accomplished by constant regimens over long periods of time (18-20). The approach has been found suitable for women surgically treated for endometrioma in order to avoid recurrence and subsequent surgery, and to preserve ovarian function and capacity. Similar to endometriosis, the adenomyosis-related symptoms (dysmenorrhea, heavy bleeding and enlarged as well as painful uterus) can be favorably influenced by oral hormonal contraceptives (21).

## 8. Myoma

Risk of myoma development was significantly reduced among post-treatment and Constant users of combined estrogen/progestogen preparations and the risk decreases with longer management (22). Constant users showed a 70% reduction of myoma size, and post-treatment users, who used the pills for seven and more years, demonstrated a 50% reduction of myoma size (23).

## 9. Pelvic Inflammatory Disease (PID)

It has been found that oral hormonal contraceptives reduce the risk of PID by 50% to 60% (24). This leads to a reduction inhospitalization days, amount of medication and operative procedures, and also the risk of ectopic pregnancy and infertility problems (3). Besides the risk reduction between 50-70%, PID occurring during the pill intake seems to be associated with less severe inflammation as judged by laparoscopy (12).

## 10. Rheumatoid Arthritis

Oral contraceptives are found to decrease the risk of rheumatoid arthritis by about 30% and in hospital-based case studies by 51% (25). In women who used oral hormonal contraceptives for more than five years the relative risk of developing mild disease was RR 0,10; 95%CI 0,01-0,06 (26). However, it appears that hormonal contraceptives do not significantly influence the long-term outcome of the disease (27).

## 11. Multiple Sclerosis

Recently it was reported that the age of first MS-symptoms was significantly higher among women who had oral hormonal contraceptives compared to women who did not (onset at 31 years versus 33 years). The age for onset of MS increased in proportion to the increased duration of OC intake, from 24 years with less than 1 year of OC intake to 31 years with more than 10 years OC intake. The women´s age at primary symptoms is significantly higher among women who gave birth before the onset of MS compared to those who did not (onset at 31 years versus 33 years) (28).

## 12. Menstrual Migraine

The basic principle in women with menstrual migraine without aura is just minimal changes in hormonal concentrations or elimination of hormonal changes all together. This can be accomplished by eliminating the standard 7- day free interval of oral hormonal contraceptive pills and taking a `long-cycle` or constant regimen (29, 30).

## 13. Bone Structure 

Preservation of bone mineral density will occur with oral hormonal contraceptives when a hypoestrogenic state is present, particularly in younger aged women, or ovarian function declines in older premenopausal women . With long-term use of oral hormonal contraceptives (˃ 5 years) protection increases in proportion to the increased duration of the use (31).

## 14. Voice

Vocal cords are hormone-dependent structures and therefore influenced by pathological changes of circulating hormone parameters such as androgens in the climacterium. In androgen excess, oral hormonal contraceptives with a combination of ethinylestradiol and a progestogen having anti-androgenic properties can lead to voice improvement or resumption. Similar positive clinical effects can be experienced in climacteric women when lack of estradiol and dominance of androgen effects may become relevant. Also, in this situation a combination of estradiol/estradiol valerate and an anti-androgenic progestogen may improve or restore the voice in professional and non-professional singers (32).

## 15. Asthma

Recent studies have indicated that oral hormonal contraceptives help to reduce asthmatic symptoms (33).

## 16. Endometrial Hyperplasia

Endometrial hyperplasia can be either prevented (for instance in women with PCOS) or successfully treated by oral hormonal contraceptives showing a progestogen dominance effect at the tissue side.

## 17. Benign Breast Disease

Many studies have shown a decreased risk of benign breast disease in OC users versus non-users. There is a statistically significant decreased risk of benign breast disease with longer duration of intake before the first full term pregnancy (24). The Royal College of General Practitioner Study has shown in 46000 women that a fixed dose of ethinylestradiol combined with increasing dose of the same progestogen (a 19 nor-testosterone derivative) lead to a decreased risk of fibroadenoma in young women (34). The Oxford Family Planning Association Study observed reduced incidences for:

1. Fibrocystic breast disease (30%),

2. Fibroadenoma (60%), and

3. Clinically found breast lumps (40%)

Diminishing the risk increases with longer duration of the use. Constant users demonstrate the lowest risk. A protection lasting up to one year after OC discontinuation was found (35). By the use of OC there was even a lower risk of breast ductual hyperplasia compared to women with no OC intake. Constant use and intake of more than eight years was associated with a lower prevalence of breast ductual hyperplasia.

## 18. Prevention of Ovarian Cancer

Risk reduction of ovarian cancer is one of the most important health benefits of oral combined hormonal contraceptives (6). The reduction of ovarian cancer is thought to be the result of suppression of ovulation which on the one hand decreases the injury and subsequent regression of the ovarian surface epithelium and on the other hand there are constant low levels of FSH and LH which also affect the ovarian surface epithelium which is the starting point of most ovarian cancers (12). The beneficial effect is already seen when combined oral hormonal contraceptives are used for as little as three to six months, although an 80% risk reduction is only achieved after intake for more than 10 years. Using combined oral hormonal contraceptives there is a 6% decrease of relative risk per year, persisting beyond 15 years of exposure. Intake before pregnancy has additional benefits (36). No difference is seen in risk reduction with different formulations of combined oral hormonal contraceptives including low dose pills (11, 37). The risk in four main histological types of epithelial ovarian cancer (serous, endometroid, mucinous, clear cell) are similarly reduced by the use of combined oral hormonal contraceptives, but not in germ cell ovarian malignancies (12). Many larger studies and meta-analyses showed similar results (38-41).

A collaborative reanalysis of worldwide data on combined oral hormonal contraceptives and ovarian cancer involving 45 epidemiologic studies with about 23000 ovarian cancer cases and about 87000 controls has demonstrated that ever use of combined oral hormonal contraceptives decreases the risk of ovarian cancer by 27%. The longer the duration of OC use the greater the risk reduction (P = 0,001) leading to a decrease of about 20% of every five years of use. The risk reduction persisted more than 30 years after combined oral hormonal contraceptives (42). From these data it was calculated that combined oral hormonal contraceptives already prevented around 200000 ovarian cancers and 100000 deaths due to the disease. Over the next decades the number of cancers prevented will rise to at least 30000 cases per year (42).

Recent data also suggest that the combined oral hormonal contraceptives can also provide primary prevention for women at risk for hereditary ovarian cancer. The adjusted odd ratio for ovarian cancer with any use of combined oral hormonal contraceptives compared to never usage was 0.5; 95% CI 0,3 -0,8. The greatest protection was observed with six and more years of intake (OR 0,3 ; 95% CI 0,1-0,7) (43). A recent case control study comparing BRCA 1 and BRCA2 mutation cases with controls demonstrated that combined oral hormonal contraceptives prevented ovarian cancer in these high-risk women (44). Combined oral hormonal contraceptives significantly reduced the risk of ovarian cancer in carriers of BRCA1 (OR 0,56 ; 95%CI 0,45-0,71) and BRCA 2 mutations (OR 0,39; 95% CI 0,23-0,66).

Women with endometriosis represent a high risk group for ovarian cancer. It was found that the use of combined oral hormonal contraceptives for more than 10 years was associated with a substantial reduction in risk among women with endometriosis (OR 0,21; 95% CI 0,08-0,58) (45).

Therefore, long-term use of combined oral hormonal contraceptives in women with endometriosis may provide substantial protection from ovarian cancer for this high-risk population because of long-term ovulation suppression (45).

## 19. Endometrial Cancer

Women using combined oral hormonal contraceptives show about a 50% reduction in the risk of endometrial cancer compared to never users, when combined oral hormonal contraceptives are used at least for one year. This protective effect increases with the duration of use and persists more than 20 or more years after discontinuation (6, 12, 31, 46). This result is true for three major histological subtypes of endometrial cancer: 1) adenocarcinoma, 2) adenosquamous carcinoma, and 3) adenoacanthoma.

Risk reduction for endometrial cancer was 80% for high-progestogen-containing pills for more than 10 years since stopping (47) and 70% for 15 to 19, and 20% for 20 and more years since stopping the use of combined oral hormonal contraceptives (39). Similar finding of RR 0,2 for more than 10 years of use was found in Sweden (48).

The subsequent use of hormone replacement therapy did not modify the long-term protecting effect of the previous use of combined oral hormonal contraceptives. Similar results were also obtained in China (48). Lower dose combined oral hormonal contraceptives appear to lead to similar results (31).

## 20. Colon Cancer

Evidence shows that combined oral hormonal contraceptives protect women from developing colon/ rectal cancer (12). Eight case control studies resulted in a pooled RR for colon/rectal cancer of 81%; 95% CI 0,69 -0,94. The pooled data estimated from cohort studies was RR 0,84; 95% CI 0,73-0,90; in all studies combined the RR was 0,82; 95% CI 0,74-0,92.

The reduction was greatest for recent use and showed no duration effect (49). Similar results were obtained by others (12, 38, 50-52). In a study by the Royal College of General Practitioners the RR for large bowel or rectal cancer was 0,72 ; 95%CI 0,58-0,90 for ever use of combined oral hormonal contraceptives (38). A recent large cohort study found a modest reduction in the risk of colon/ rectal cancer (RR 0,83 ; 95% CI 0,73-0,94), but no trend was seen for RR with increased duration of the use of combined oral hormonal contraceptives.

To illustrate oncological non-contraceptive use of combined oral hormonal contraceptives it was recently pointed out that the use of combined oral hormonal contraceptives by nuns would protect them from oncological hazards of nulliparity (53).
